# Prevalence, Distribution, and Host Range of *Peste des petits ruminants virus*, Turkey

**DOI:** 10.3201/eid0807.010471

**Published:** 2002-07

**Authors:** Aykut Özkul, Yilmaz Akca, Feray Alkan, Thomas Barrett, Taner Karaoglu, Seval Bilge Dagalp, John Anderson, Kadir Yesilbag, Can Cokcaliskan, Ayse Gencay, Ibrahim Burgu

**Affiliations:** * Ankara University, Ankara, Turkey; † Institute for Animal Health, Pirbright Laboratory, Surrey, United Kingdom

**Keywords:** Morbillivirus, Peste des petits ruminants virus, rinderpest, seroprevalence, reverse transcription-polymerase chain reaction, molecular epidemiology

## Abstract

*Peste des petits ruminants virus* (PPRV, genus *Morbillivirus*), which causes a severe disease in sheep and goats, has only recently been officially declared to be present in Turkey. We carried out a study to determine the prevalence, distribution, and host range of PPRV in Turkey. A total of 1,607 animals, reared in 18 different locations, were monitored for the presence of antibodies to PPRV and the related virus of large ruminants, *Rinderpest virus* (RPV). Only two farms had animals that were free of antibody responses to either disease. Prevalence for PPRV infection varied (range 0.87%–82.6%) and was higher in sheep (29.2%) than in goats (20%). The overall antibody responses to PPRV and RPV were 22.4% and 6.28%, respectively. Two PPRVs of lineage 4, which comprises many other PPRVs whose origins are in the Middle East, the Arabian Peninsula, and southern Asia, were isolated from Turkish sheep.

*Peste des petits ruminants virus* (PPRV) is a morbillivirus that primarily infects sheep and goats. The virus is present in Africa (1–3), the Middle East (4), the Arabian Peninsula (5), and southern Asia (6,7) and is closely related to *Rinderpest virus* (RPV), *Canine distemper virus,* and human measles virus (8). Infection with PPRV results in an acute, highly contagious disease characterized by fever, anorexia, necrotic stomatitis, diarrhea, purulent ocular and nasal discharges, and respiratory distress (9,10). Infection rates in sheep and goats rise with age, and the disease, which varies in severity, is rapidly fatal in young animals (10,11). As with other morbillivirus infections, PPRV needs close contact between infected and susceptible animals to spread (10). The two ruminant morbilliviruses, PPRV and RPV, have common antigens demonstrable in a variety of serologic test systems, and they also show a degree of cross-neutralization (12,13). Originally PPRV was considered a variant of RPV adapted to small ruminants; however, the two viruses have separate epizootiologic cycles in nature, and each exists in its own right (14,15).

PPRV infection has only recently been officially reported in Turkey, in September 1999 (16,17), but some reports indicate it was present before then (18,19). The objectives of our research were to determine the seroprevalence of PPRV infection in cattle, sheep, and goats; determine the regional distribution of PPRV in Turkey; isolate and characterize the Turkish virus; and compare its genome sequence with those of other PPRV sequences in the sequence database maintained at the World Reference Laboratory, Pirbright, United Kingdom.

## Materials and Methods

### Animals Used in the Study

Domestic ruminant species (cattle, sheep, and goats) from throughout Turkey were examined for virus-specific antibodies. The sampling procedure depended on the presence of suspected infection and focused on two groups of animals. The first included 193 sheep that local authorities reported as having clinical signs of PPRV infection. These animals were examined, blood samples were collected, and any animals with signs of disease were sampled by swabbing for virus isolation. Cattle grazing with sheep or goats were also sampled to monitor for antibodies to the two viruses. The second group consisted of 1,414 animals randomly selected for serologic screening for PPRV and RPV antibodies from herds near the flocks of sheep and goats in which PPRV-like infection was reported. The numbers of serum samples collected from sheep, goats, and cattle were 884, 209, and 321, respectively.

### Tests for PPRV- and RPV-Specific Antibodies

Competitive enzyme-linked immunosorbent assays were performed as described in the manual of Peste des Petits Ruminants enzyme-linked immunosorbent assay (ELISA) kit (20) and the Office International des Epizooties Manual of Standards (9). Each serum sample, regardless of the species from which it was obtained, was tested for the presence of antibodies to RPV and PPRV.

### Virus Isolation Material and Infection of Cell Cultures

A total of 328 field samples, including heparinized blood, organ (lung), and swab specimens, were cultured to obtain virus isolates. The processed samples were spread onto Vero cells seeded in rolling culture tubes. The cells were grown in Dulbecco’s modified Eagle medium enriched with 5% fetal bovine serum as a regular culture medium. The cell culture media were changed every 2 days and the inoculated cells observed for 12–14 days. The positive culture tubes were frozen at -80°C when the cytopathic effect (CPE) was 90%, and virus stocks were prepared from the positive samples.

### Detection of PPRV RNA

Detection of PPRV RNA by reverse transcription-polymerase chain reaction (RT-PCR) was performed as described (21). PCR amplification was carried out by with a PPRV-specific primer set (PPRVF1b: 5´AGTACAAAAGATTGCTGATCACAGT and PPRVF2d: 5´GGGTCTCGAAGGCTAGGCCCGAATA) selected from the F protein gene sequence, which is expected to amplify a 448-bp DNA product. RT-PCR products were digested by using *Eco*RI at 37°C for 1 hour. Samples were then analyzed on 1.7% agarose gels to determine the cleavage patterns of the amplicons. DNA products obtained with PPR F1b and F2d primers were sequenced by using a T7 polymerase-based commercial kit (Pharmacia Diagnostics AB, Uppsala, Sweden) with ^35^SdATP as the radiolabel.

### Phylogenetic Analysis

Sequence data were analyzed with the GCG (Genetics Computer Group Inc., Madison, WI) package. The nucleic acid sequences obtained from PCR products were aligned with known sequences from representatives of the *Morbillivirus* genus, and the phylogenetic tree was generated with the DNADIST and FITCH programs of the PHYLIP 3.73 software (22).

## Results

### Clinical Findings

Animals with clinical signs of PPRV were detected in 11 provinces (Table 1). In many cases, inspections of flocks confirmed PPRV-suspect cases reported by local veterinarians or identified symptoms indicative of PPRV infection. Most clinical cases were characterized by excessive oculonasal discharge, mild ulcerative stomatitis, dyspnea, and coughing. Severe mucosal eruptions and intestinal signs were not detected.

**Table 1:** *Peste des petits ruminants virus* (PPRV*)*–specific antibody prevalence in animals with clinical symptoms indicative of PPRV, Turkey

Location	Animal	Animals with PPRV-suspected symptoms		
		No.	PPRV positive	%
Batman	Sheep	8	7	87.5
Denizli	Goat	10	6	60.0
Cihanbeyli	Sheep	8		
Amasya	Sheep	20		
Sakarya	Sheep	19	3	15.8
Eskisehir	Sheep	5	4	80.0
Malatya	Sheep	3	2	66.6
Sivas	Sheep	23	6	26.0
Isparta	Sheep	32	32	100.0
Aydin	Sheep	42	10	23.8
Van	Sheep	43	40	93.0
Total		213	110	51.6

### Serologic Status of Sampled Animals

**Figure 1:**
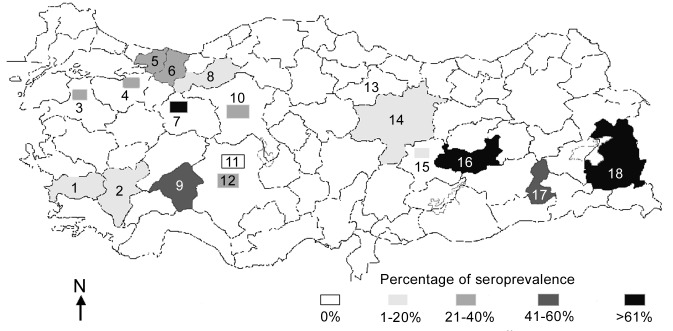
Areas of Turkey sampled to detect the presence of infection with *Peste des petits ruminants virus* and *Rinderpest virus.* Numbers in parentheses indicate the number of serologic test materials collected from each location. Rectangles indicate a single outbreak; shaded provinces had multiple outbreaks. Key: 1, Aydin (100); 2, Denizli (164); 3, Balikesir (40); 4, Bursa (40); 5, Kocaeli (100); 6, Sakarya (100); 7, Eskisehir (5); 8, Bolu (160); 9, Isparta (100); 10, Ankara (20); 11, Cihanbeyli (75); 12, Konya (50); 13, Amasya (20); 14, Sivas (109); 15, Malatya (3); 16, Elazig (272); 17, Batman (50); 16, Van (199).

A total of 1,607 animals from 18 farms were sampled for antibodies to PPRV and RPV (Figure 1). Only two farms (Cihanbeyli and Amasya) had no animals with antibodies specific to either virus. The overall percentages of antibody response to PPRV and RPV were 22.4% and 6.28%, respectively (Table 2). Prevalences of PPRV infection varied between flocks, ranging from 0.87% to 82.60%; however, these figures may not be accurate because of the small sample sizes. In general, the level of PPRV infection was higher in sheep; however, the highest seroprevalence (82.6%) was found in goats in Sakarya Province, where two PPRV isolates were identified from sheep during this project. Of 1,077 sheep examined, 315 (29.2%) were seropositive for PPRV and 1.2% for RPV. The 10 RPV-seropositive sheep in Bursa Province (in a flock with no clinical PPRV) were reported to have been vaccinated against RPV**,** while only 1 sheep in Konya was found to have seroconverted, probably following natural infection with RPV before 1999. The overall occurrence of PPRV infection in cattle was 15.57% (a total of 50 animals), and approximately 27% of cattle were antibody positive for RPV, indicating previous exposure to the virus either by natural infection or, most probably, by vaccination, since all cattle in the study were >6 months of age.

**Table 2:** Analysis of antibody response against *Rinderpest virus* (RPV) and *Peste des petits ruminants virus* (PPRV*),* by species, Turkey

Species	Yr of sampling	No. of sera	No. (%) of animals with antibodies to	
			PPR	RPV
Sheep	1999–2000	1,077	315 (29.20)	13 (1.20)
Goats	1999	209	42 (20.00)	1 (0.47)
Cattle	1999–2000	321	3 (0.90)	87 (27.10)
Total		1,607	360 (22.40)	101 (6.28)

The study showed no substantial relationship between the occurrence of PPRV infection and geographic location. Although the main portal of entry of the disease is thought to be in the southeastern part of Anatolia, distribution of the prevalence values did not show a clear pattern across the country, and the disease was detected in varying percentages in almost every region studied (Figure 1).

### Virus Isolation

**Figure 2:**
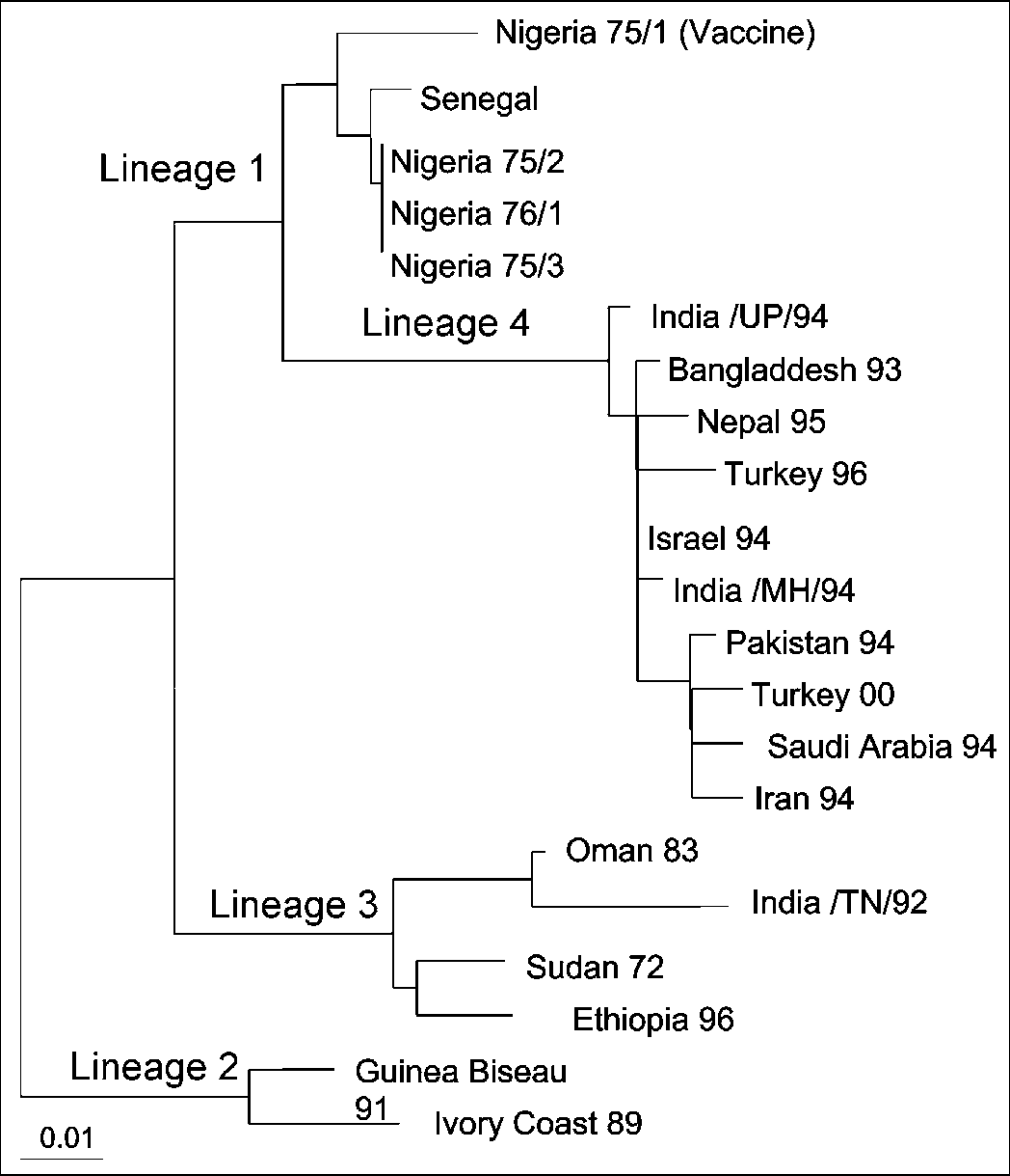
Phylogenetic relationship of the Peste des petits ruminants viruses isolated in Turkey in 2000 to other virus isolates. The tree is based on partial sequence data from the fusion (F) protein gene (7) and was derived by using the PHYLIP DNADIST and FITCH programs (22). Branch lengths are proportional to the genetic distances between viruses and the hypothetical common ancestor at the nodes in the tree. The bar represents nucleotide substitutions per position.

A total of 328 samples were spread onto Vero (African Green Monkey Kidney) cells. Two nasal swab samples (Sakarya 1 and Sakarya 2), from sheep in Sakarya Province, showed CPE on Vero cells. The CPE was observed on day 3 after inoculation and was initially characterized by the formation of rounded cells; later, syncytia developed. RT-PCR was performed on cell culture supernatants after the first passage on the Vero cells. The expected amplification product of 448 bp was observed by using RNA prepared from culture supernatants from only the two samples (data not shown). Restriction fragment length polymorphism analysis of the RT-PCR products indicated nucleotide substitutions in the *Eco*RI recognition sequence site in the amplified genome region of the isolates. While the PPR vaccine strain (Nigeria 75/1) produced, as expected, two fragments of 202 bp and 246 bp on cleavage with *Eco*RI, isolates Sakarya 1 and Sakarya 2 were not digested by this restriction enzyme (data not shown). Partial sequencing of the F protein–coding region of the two PPRV isolates showed them to be identical (GenBank accession number AF384687). The Turkey 2000 sequence was then aligned with the sequences of other PPRV isolates from around the world. Figure 2 shows the inferred phylogenetic relationship between the isolates recovered in this research and other PPRVs. The Turkish isolates belonged to PPRV lineage 4 (7), which originates in the Middle East, Arabia, and southern Asia.

## Discussion

We investigated the prevalence, host range, and distribution of PPRV in small private farms in Turkey. We also demonstrated the presence of the disease by observing animals in the field and by isolating virus from clinical specimens. This wide-ranging survey is the first to be carried out on this disease in Turkey. PPRV infection has only recently been officially declared to be present in Turkey in the Elazig Province in eastern Anatolia (16,17). Our research provided valuable data on the serologic status of the three domestic ruminant species (cattle, sheep, and goats) with respect to PPRV. Infection with PPRV was demonstrated in 16 of 18 farms we sampled (except for Cihanbeyli and Amasya). On a flock basis, the highest virus prevalence (82.6%) was in goats in Sakarya, where two isolates were identified from sheep. The second highest prevalence (80%) was in sheep in Eskisehir, followed by 72% in sheep in Van Province and 66.6% in sheep in Malatya Province. Van and Malatya Provinces are in southeastern Turkey near the Iranian border; the remaining provinces are mainly in central Anatolia (Figure 1). Variation in prevalence is probably related to the intensity of trade of illegally imported small ruminants (23).

The prevalence of the disease was as high as 28.5% in sheep and goats reared in small private flocks, and the disease was found in almost every region across Turkey. Occurrence of infection did not vary substantially by geographic locations of the livestock tested. Although the presence of PPRV infection in Turkey has been reported before (18,19), the impact of the disease on production of livestock animals has not previously been investigated. The overall prevalence of PPRV was 22.4% of the ruminant population. These results indicate much lower prevalence than the 88.3% reported by Tatar (19). However, if the overall percentage of PPRV infection takes into account animals reported as having clinical signs, the level increases to 51.6% (Table 1). Another ruminant morbillivirus infection, RPV in cattle, caused great economic losses from the deaths or slaughter of affected (or suspected infected) animals in Turkey in recent years (24). Because PPRV and RPV are antigenically related, the attenuated RPV vaccine has been used to protect small ruminants against PPRV. According to anecdotal reports from the field, veterinarians and animal owners widely used the RPV vaccine to protect small ruminants against PPRV infection in some parts of Turkey before RPV vaccination was stopped in the year 2000. This might be one reason for the lower percentage of PPRV-positive animals found in this study.

Cattle act as dead-end hosts for PPRV and show no clinical signs of infection. Nevertheless, they develop a humoral immune response to PPRV that protects them against natural or experimental challenge with virulent RPV (12). In our study, the percentage of natural PPRV infection in cattle was low (0.9%), and all these were in cattle that had contact with infected sheep flocks. Cattle seropositive for PPRV could cause confusion when monitoring for antibodies to RPV after a vaccination campaign to eradicate RPV. Natural infection of cattle with PPRV might prevent the immune response to the RPV vaccine because the PPRV-specific antibodies could neutralize the live attenuated vaccine virus. The cattle would still be protected from subsequent RPV challenge by heterologous PPRV antibody but would register as seronegative when tested in the RPV competitive ELISA. This false value could lead to a low estimation of herd immunity to RPV or suggest that the vaccination coverage was inadequate (12). This risk is high in mixed breeding systems, such as the small-scale production units common in rural regions of Turkey. On the other hand, 14.6% of the cattle had antibodies to both viruses (PPRV and RPV)**.** This finding may indicate that in some cases no interference occurs. Another possible reason is the cross-reactivity of the PPRV assay for antibodies to RPV, as noted by Anderson et al. (25).

Only two PPRVs were isolated—from nasal swab samples of two sheep from Sakarya Province. The reason for the poor success in isolating virus could be the nature of the samples. In previous studies, virus isolations were made from spleen, mesenteric lymph nodes (7,26), or intestinal epithelial smears (15) collected during necropsy of affected animals by inoculation on to Fetal Lamb Kidney (FLK) (19,26) or Vero (15,19) cells. In our study, however, most samples were taken from surviving animals that may have been past the clinical phase of the disease, when the virus is secreted, and so were not likely to yield virus isolates from swabs. Moreover, owners of many sick animals did not grant permission to euthanize them, so internal organs were not available for gross pathologic analysis in most cases. According to a previous study (19), FLK and Vero cells are equally susceptible to PPRV; thus, the use of Vero cells was probably not a factor in our poor success with virus isolation.

Our use of molecular epidemiologic techniques provided data that suggest cross-border transmission into Turkey of PPRV infection that is actively circulating in neighboring countries. The viruses we isolated are PPRV lineage 4, which includes viruses whose origins are in the Middle East, Arabia, and south Asia (7). Because of its geographic location, Turkey has borders with countries where many economically important infectious diseases are endemic. Thus, one of the neighboring countries in the Middle East region is most likely the source of infection. Since the terrain of eastern and southeastern Anatolia permits uncontrolled animal movement, restricting the spread of infectious diseases into the country has been difficult. Therefore, the importance of PPRV as a threat to livestock should be considered, together with other economically important diseases, and measures taken to prevent the import and subsequent spread of such diseases.

## References

[1] Taylor WP. The distribution and epidemiology of peste des petits ruminants. Prev Vet Med. 1984;2:157–66. 10.1016/0167-5877(84)90059-X

[2] Awa DN, Njoya A, Ngo Tama AC. Economics of prophylaxis against peste des petits ruminants and gastrointestinal helminthosis in small ruminants in north Cameroon. Trop Anim Health Prod. 2000;32:391–403. 10.1023/A:1005233703331 11147279

[3] Roeder PL, Abraham G, Kenfe G, Barrett T. Peste des petits ruminants in Ethiopian goats. Trop Anim Health Prod. 1994;26:69–73. 10.1007/BF02239901 7941031

[4] Lefevre PC, Daillo A, Schenkel S, Hussein S, Staak G. Serological evidence of peste des petits ruminants in Jordan. Vet Rec. 1991;128:110. 2024417 10.1136/vr.128.5.110

[5] Abu-Elzein EME, Hassanien MM, Al-Afaleq AI, Abdelhadi MA, Honsawai FMJ. Isolation of peste des petits ruminants from goats in Saudi Arabia. Vet Rec. 1990;27:309. 2238415

[6] Shaila MS, Purushothaman V, Bhavasar D, Venugopal K, Venkatesan RA. Peste des petits ruminants of sheep in India. Vet Rec. 1989;125:602. 2609485

[7] Shaila MS, Shamaki D, Forsyth M, Diallo A, Goatley L, Kitching P, Geographic distribution and epidemiology of peste des petits ruminants viruses. Virus Res. 1996;43:149–53. 10.1016/0168-1702(96)01312-3 8864204

[8] Barrett T. Morbilliviruses: dangers old and new. In: Smith GL, McCauley JW, Rowlands DJ. New challenges to health: the threat of virus infection. Society for General Microbiology, Symposium 60. Cambridge: Cambridge University Press; 2001. p. 155–78.

[9] Office International des Epizooties (OIE). OIE manual of standards for diagnostic tests and vaccines. List A and B diseases of mammals, birds and bees. Paris: The Office; 2000.

[10] Lefevre PJ, Diallo A. Peste des petits ruminants. [OIE]. Rev Sci Tech. 1990;9:951–65. 10.20506/rst.9.4.532 2132714

[11] Wosu LO. Current status of peste des petits ruminants (PPR) disease in small ruminants—a review article. Stud Res Vet Med. 1994;2:83–90.

[12] Anderson J, McKay JA. The detection of antibodies against peste des petits ruminants virus in cattle, sheep and goats and the possible implications for rinderpest control programmes. Epidemiol Infect. 1994;112:225–31. 10.1017/S0950268800057599 8119361 PMC2271469

[13] Taylor WP. Protection of goats against peste des petits ruminants with attenuated rinderpest virus. Res Vet Sci. 1979;27:321–4. 542719

[14] Dardiri AH, De Boer CJ, Hamdy FM. Response of American goats and cattle to peste des petit ruminants.Vet Lab Diagn 1976;337–44.

[15] Taylor WP, Abegunde A. The isolation of peste des petits ruminants virus from Nigerian sheep and goats. Res Vet Sci. 1979;26:94–6. 472495

[16] Emergency Prevention System (EMPRES) for Transboundary Plant and Animal Pests and Diseases 2000; no. 13. Available from: URL://www.fao.org/empres

[17] Office International des Epizooties (OIE). OIE disease information 1999;12:137.

[18] Alcigir G, Vural SA, Toplu N. Türkiye'de kuzularda peste des petits ruminants virus enfeksiyonunun patomorfolojik ve immunohistolojik ilk tanimi. Ankara Universitesi Veteriner Fakültesi Dergisi. 1996;43:181–9.

[19] Tatar N. Koyun ve keçilerde küçük ruminantlarin vebasi ve sigir vebasi enfeksiyonlarinin serolojik ve virolojik olarak arastirilmasi [dissertation]. Ankara: Ankara Üniversitesi, Saglik Bilimleri Enstitusu; 1998.

[20] Peste des Petits Ruminants ELISA kit, Competitive Enzyme Immunoassay for Detection of Antibody to PPR Virus. Bench protocol, version PPR 1.0, January 1993. Joint FAO/IEAE Programme, Animal Production and Health. Pirbright, United Kingdom: World Reference Laboratory for Rinderpest; 1993.

[21] Forsyth M, Barrett T. Evaluation of polymerase chain reaction for the detection of rinderpest and peste des petits ruminants viruses for epiemiological studies. Virus Res. 1995;39:151–63. 10.1016/0168-1702(95)00076-3 8837881

[22] Felsenstein J. PHYLIP – Phylogeny inference package. Cladistics. 1989;5:164–6.

[23] Al-Naeem A, Abu-Elzein EME, Al-Afaleq AI. Epizootiological aspects of peste des petits ruminants and rinderpest in sheep and goats in Saudi Arabia. [International Office of Epizooties]. Rev Sci Tech. 2000;19:855–8. 11107629 10.20506/rst.19.3.1261

[24] Burgu I, Akca Y, Ozkul A. Rapid detection of rinderpest virus antigen using pig-anti-CDV-PO conjugate in cell culture. In: Proceedings of International Symposium on Morbillivirus infections. 12–13 June 1994, Hannover Veterinary School, Germany. Hannover: European Society for Veterinary Virology; 1995.

[25] Anderson J, McKay JA, Butcher RN. The use of monoclonal antibodies in competitive ELISA for the detection of antibodies to rinderpest and peste des ruminants viruses. In: Jeggo MH, editor. The sero-monitoring of rinderpest throughout Africa phase one. The proceedings of a final research co-ordination meeting of the FAO/IAEA/SIDA/OAU/IBAR/PARC Co-ordinated Research Programme. Ivory Coast. IAEA-Techdoc 1990;623:43–53.

[26] Furley CW, Taylor WP, Obi TU. An outbreak of peste des petits ruminants in a zoological collection. Vet Rec. 1987;121:443–7. 3424615 10.1136/vr.121.19.443

